# Aligning everyday life priorities with people’s self-management support networks: an exploration of the work and implementation of a needs-led telephone support system

**DOI:** 10.1186/1472-6963-14-262

**Published:** 2014-06-17

**Authors:** Christian Blickem, Anne Kennedy, Praksha Jariwala, Rebecca Morris, Robert Bowen, Ivaylo Vassilev, Helen Brooks, Tom Blakeman, Anne Rogers

**Affiliations:** 1 NIHR Collaboration for Leadership in Applied Health Research and Care (CLAHRC) Greater Manchester, Centre for Primary Care, Institute of Population Health, University of Manchester, Oxford Road, Manchester M13 9PL, UK; 2 NIHR Collaboration for Leadership in Applied Health Research and Care (CLAHRC) Wessex Health Sciences, University of Southampton, Highfield Campus, 12 University Road, Southampton SO17 1BJ, UK

**Keywords:** Vascular disease, Self-management, Long-term conditions, Normalization process theory, Randomized controlled trial, Inequalities, Social networks, Social prescribing, Chronic kidney disease, Asset-based community development

## Abstract

**Background:**

Recent initiatives to target the personal, social and clinical needs of people with long-term health conditions have had limited impact within primary care. Evidence of the importance of social networks to support people with long-term conditions points to the need for self-management approaches which align personal circumstances with valued activities. The Patient-Led Assessment for Network Support (PLANS) intervention is a needs-led assessment for patients to prioritise their health and social needs and provide access to local community services and activities. Exploring the work and practices of patients and telephone workers are important for understanding and evaluating the workability and implementation of new interventions.

**Methods:**

Qualitative methods (interviews, focus group, observations) were used to explore the experience of PLANS from the perspectives of participants and the telephone support workers who delivered it (as part of an RCT) and the reasons why the intervention worked or not. Normalisation Process Theory (NPT) was used as a sensitising tool to evaluate: the relevance of PLANS to patients (coherence); the processes of engagement (cognitive participation); the work done for PLANS to happen (collective action); the perceived benefits and costs of PLANS (reflexive monitoring). 20 patients in the intervention arm of a clinical trial were interviewed and their telephone support calls were recorded and a focus group with 3 telephone support workers was conducted.

**Results:**

Analysis of the interviews, support calls and focus group identified three themes in relation to the delivery and experience of PLANS. These are: *formulation of ‘health’ in the context of everyday life; trajectories and tipping points: disrupting everyday routines; precarious trust in networks.* The relevance of these themes are considered using NPT constructs in terms of the work that is entailed in engaging with PLANS, taking action, and who is implicated this process.

**Conclusions:**

PLANS gives scope to align long-term condition management to everyday life priorities and valued aspects of life. This approach can improve engagement with health-relevant practices by situating them within everyday contexts. This has potential to increase utilisation of local resources with potential cost-saving benefits for the NHS.

**Trial registration:**

ISRCTN45433299.

## Background

### Everyday life and living with long-term health problems: the limitations of traditional self-management support

Whilst self-management support is widely advocated it is clear that existing strategies are of limited benefit because they fail to take account of everyday circumstances of patients and the range of work required to manage their health within the context of their daily lives [1-3]. Self-management support is frequently disconnected from the realities of social deprivation and the mundane everyday demands of living life with a long-term condition (LTC) are overlooked as are the capacity and personal support needed to balance everyday life practicalities with the additional work required to manage a LTC [4]. Furthermore, the focus of self-management support for LTC management tends to be on moments of crisis or the temporary and transient, and lack engagement with a wider set of resources and networks [1,5].

These approaches have failed to incorporate the broader focus on creating and developing healthy and sustainable communities advocated for tackling inequalities and supporting long-term condition management. [6,7]. The latter implicates the inclusion of engagement with the third sector and the mobilisation of community resources to meet patients’ needs. This acknowledges the value of achieving a wider set of goals such as returning to work or living independently and meeting the additional needs that can impact on a person’s total health and well-being [8].

Asset-Based Community Development (ABCD), referrals facilitator schemes and social prescribing have all been tried as alternative ways to support people with long-term health problems by engaging with and mobilising community resources [9-14]. However these have had limited impact within health services and have not focussed specifically on engaging and linking networks and resources as a response to patient defined need.

As part of a Randomised Controlled Trial aimed at managing chronic kidney disease in primary care, a needs-led intervention to improve networks of support for people with long-term health problems, Patient-Led Assessment for Network Support (PLANS) was developed [15]. PLANS was designed to address the problem of engagement with and the mobilisation of community resources to support people with generic long-term health problems and can be used to prioritise people’s own health and social needs in a way which tailors access to local community services [16]. Delivery of PLANS for the BRIGHT trial and Summary of the PLANS support calls conducted for the BRIGHT trial sections elucidate how PLANS was delivered in the trial. While PLANS was developed with the underlying assumption that chronic illness management and broader well-being are closely intertwined in people’s everyday lives, it was also assumed that sustainable behaviour change and engagement with available resources could only be feasible by putting the emphasis on what is acceptable to people.

In summary, PLANS aims to:

•increase social contact and promote community support and engagement.

•create awareness of and link people to available community support and local health relevant resources.

•be based on need (clinical or social) and personal preference.

#### Understanding the work involved with delivering PLANS

The effective delivery of a self-management support intervention such as PLANS goes beyond the supply of information and the nature of the work involved requires people to proactively seek support or attend meetings or classes. This work may require the support of family members or friends to provide transport or an appropriate other to take over any roles on their behalf, e.g. caring for a partner or grandchildren, to allow the uptake of the PLANS options.

#### Study aims

The BRIGHT trial led to significant improvement in health outcomes. These results will be reported in more detail elsewhere. Therefore this qualitative study was designed to understand the active ingredients of the intervention to aid generalizability and facilitate translation into everyday practice by a) understanding the ‘work’ required of participants and telephone support workers and the skills required to effectively deliver and engage with PLANS; b) understanding who it works for and why and what contexts make successful implementation more likely; c) exploring the experience of PLANS and the processes of delivery and engagement from the perspectives of participants and the telephone support workers who delivered it; and d) to gain insight into the perceived relevance of PLANS to patients who received it and determine how patient-directed resources are implemented in people’s everyday lives.

This study was conducted as part of the NIHR Collaboration for Leadership in Applied Health Research and Care for Greater Manchester (GM CLAHRC), which is a coordinated programme of research that aims to create, adapt and implement strategies for health services and health professionals to support socially disadvantaged people with long-term vascular conditions. A focus of GM CLAHRC is on the need for implementation and translation outside the NHS which recognises the critical role played by personal communities, local and community groups, health and non-health professionals, as well as people with LTCs for effective and sustainable ways to improve long-term condition management [3]. The PLANS tool is designed to provide alternative ways for health services and professionals to support people with LTCs and implement self-care strategies by mobilising community resources and informal networks of support.

#### The Bringing Information and Guided Help Together (BRIGHT) trial

The BRIGHT trial is a multi-site, longitudinal patient-level randomised controlled trial which aims to implement and evaluate the clinical and cost-effectiveness of a self-management intervention involving an information guidebook, tailored access to local resources and telephone support for people with stage 3 Chronic Kidney Disease. The BRIGHT intervention comprised a package of support which included:

1. A kidney information guidebook.

2. A PLANS booklet and access to an interactive website with tailored access to local resources.

3. A PLANS telephone support from a dedicated telephone support worker.

The primary outcome measures are self-management capacity, health-related quality of life and blood pressure control compared to care as usual. A total of 436 patients with an existing diagnosis of stage 3 CKD from 24 GP practices in the Greater Manchester area were recruited between April and November 2012. Inclusion criteria were a diagnosis of stage 3 CKD (stage 3a or 3b) as recorded on the practice’s CKD register, plus attendance at a recent routine disease review appointment (maximum 8 weeks prior to recruitment). Participants who were unable to communicate in English, lacked capacity to provide informed consent, or were in receipt of palliative care were excluded. Only one person per household was eligible to take part, to avoid potential contamination across trial arms. Detailed methods are reported in the trial protocol [15].

Chronic kidney disease (CKD) is a developing area of research [17]. CKD is growing in prevalence and can lead to cardiovascular morbidity and mortality which has led to clinical guidance highlighting the importance of early identification and active management of CKD to maintain vascular health in primary care [18]. CKD is categorised into five stages with stage 5 indicating renal failure. Stages 1 to 3 are managed in primary care and are common with around 5% of the population having early stage kidney disease. There is little or no specific support or information for CKD stage 3 but there is great potential for avoiding future health problems if managed more effectively. Chronic kidney disease often exists with other conditions such as hypertension, diabetes and ischaemic heart disease and is associated with low socioeconomic status [19,20].

#### Delivery of PLANS for the BRIGHT trial

Seven telephone support workers received training and a training manual was provided. The support calls covered the following topics:

•Gaining background information about participants’ living situation.

•Ascertaining how participants were coping with their health.

•The degree of satisfaction with current activities and the support they receive.

•Participants hobbies and interests.

•Current and previous groups/services/activities attended or used.

Participants were taken through the online PLANS questionnaire by a telephone support worker and then offered a set of results of local activities and services linked to their identified needs (this questionnaire is detailed in another paper [16]). Brief descriptions of these results were given to the patient who was asked if they were interested in any further information about any of the groups, activities or services. The telephone support workers were guided by the expressed needs of participants, their personal preferences and the background information they were provided with. A follow up call one month later was conducted to identify any further information needs and an opportunity to go through the PLANS questionnaire again.

#### Summary of the PLANS support calls conducted for the BRIGHT trial

The telephone support calls were conducted by 7 telephone support workers. All patients received the kidney information guidebook and PLANS booklet. In total 207 support calls were made (94.5% of patients in the intervention arm).

##### Declines

Four patients could not be contacted. Three patients elected not to have a telephone support call and reasons were not given. Three patients withdrew from the trial before or at the initial phone call. One patient passed away before the initial phone call could be completed. Three participants elected not to complete the PLANS questionnaire during their support call. Two patients felt PLANS was of no relevance to them and one patient was hard of hearing and whose wife took the call on his behalf, and refused to answer the questionnaire.

##### Baseline support call

During the telephone support call patients were asked to complete the PLANS questionnaire and then the telephone support workers gave patients their results. Patients were then asked to select services/groups/information which were of interest to them and relevant information was sent to patients after the call. Additional file 1 provides a summary of the top 10 services/groups/information sent to patients after the telephone support call. The most popular piece of information was a healthy eating guide for people with kidney problems which was requested by 42 patients. Four of the top ten services/groups/information were kidney-related. Also popular were resources for older people [4] and community centres [2].

##### One month follow up call

The follow-up support call was intended to give patients support about making contact with groups/services/information they were interested in or to offer further information. 202 (97.6%) follow-up calls were successfully completed one month after the first support call. Two patients did not want to receive a follow-up call for which no reasons were given. Telephone support workers were unable to contact three patients at one month follow-up. Overall the number of patients that used/intended to use information/services as a result of the telephone consultation at 1 month was 48 (23.5%).

## Methods

The methods of recruitment to the BRIGHT trial are described in Blickem et al [15]. 20 patients in the intervention arm of the BRIGHT trial were interviewed and their telephone support calls were recorded. These included 15 women and 5 men. At the baseline assessment, participants in the intervention arm of the trial were invited to take part in the current study which involved the audio recording of the telephone support calls (baseline and follow up) and a qualitative interview. Following the delivery of the telephone support call, a convenience sample of participants who consented to be contacted for this qualitative study were contacted by a researcher to arrange an interview within two weeks of delivery of the telephone support call. At the interview informed consent was obtained to conduct and audio record the interview. The length of interviews varied from approximately 40 to 90 minutes.

A maximum variation sample based on age, gender and ethnicity was attempted for this qualitative study, hence we recruited patients that represented the spectrum of ages represented in the BRIGHT trial (youngest 48 years to oldest 90 years).

Criteria for inclusion was also based on the varying degrees of engagement with PLANS as ascertained from regular bi-monthly meetings with the telephone support workers. At these meetings the telephone support workers gave summaries of participants who had received the intervention and participants who broadly fell into one of two groups of ‘engaged’ and ‘not-engaged’ were identified. A fairly even distribution of both groups was recruited to this study. Three researchers CB, RB and HB all with health sciences backgrounds with extensive experience of qualitative interviewing conducted the interviews. CB led the development of PLANS and was a principle investigator for the BRIGHT trial. A focus group with three of the telephone support workers was conducted post-trial by CB and RM which lasted approximately 90 minutes.

A first draft of the topic guide was developed by all authors at early team meetings. After the first round of six interviews, summaries of these interviews were shared with the research team at a team meeting. It was then agreed to add the following recurring theme from the interviews: ‘What is the influence of current and previous engagement with activities on the uptake of PLANS recommendations?’. Topics covered during the interviews and focus groups can be found in Additional file 2. All calls, interviews and the focus group were recorded and transcribed by an external agency.

### Ethical approval

This study received full ethical approval from the Health Research Authority (REC reference: 11/NW0855).

### Analysis

Normalisation Process Theory (NPT) was used as a sensitising tool to explore the processes of delivery and engagement [21,22]. We considered that Normalization Process Theory would be a useful analytical tool because NPT is a robust theory of implementation that helps provide awareness of the work involved in embedding and sustaining practices associated with an intervention, and thus aids understanding of what becomes normalized into everyday settings. NPT was developed to understand the embedding of new technologies into health systems PLANS is such a technology and our focus was on the work that patients and support workers needed to do to ensure the effectiveness of PLANS in accessing better support. NPT is divided into four constructs which were used to evaluate: the relevance of PLANS to patients who received it (coherence); the processes of engagement and buy-in (cognitive participation); the work done to enable PLANS to happen and who this implicates (collective action); the perceived benefits and costs of PLANS (reflexive monitoring) [21,23,24]. All authors contributed to the analysis. A coding framework was developed by CB and AK which was informed by NPT (Additional file 3) and a first round of analysis was conducted using this framework. It was agreed that the analysis should also be inductive and to examine data that did not appear to ‘fit’ with the chosen theoretical framework so that important concepts or themes were not missed. All transcripts were each analysed and coded by both the first author and one of the other authors. After this round of analysis all authors attended two meetings to discuss and reach consensus about the consistency of the coding and share emergent themes from the analysis. After consensus was agreed on themes and codes a second round of analysis was conducted. CB with AK wrote the first draft of the paper and co-authors provided feedback on iterations.

## Results

The average age of this study sample was comparable with the trial (68.95 years compared with 72.1 years for the trial). The percentage of females in this study (75%) was higher than the trial (58.5%). See additional file 4 for patient demographics.

Using the four constructs of NPT Table 1 summarises the thinking and work needed by participants and telephone support workers for the successful implementation of PLANS.

**Table 1:** Summary of the work involved with PLANS

**NPT component**	**Participants**	**Telephone support workers**
**Coherence**		
Sense-making	Understand the relevance to health of social engagement, practical support and wellbeing	Understand the role of social networks support in health
**Cognitive participation**		
Buy-in and engagement	See the relevance of PLANS and link with their needs or preferences	See the worth of working with patients to assess their needs
**Collective action**		
The work of putting PLANS into operation	Assess their own needs, Discuss their preferences.	Attend training – learn skills to communicate the importance of social, practical and wellbeing dimensions of health management
	Reflect on past activities	Relate PLANS with personal histories, needs, and stated preferences of patients
	Contact community resource, get there, find others to step in and take on responsibilities, attend group, class	Find relevant and acceptable community resources and provide clear information to participants
		Motivate
**Reflexive monitoring**		
Appraisal of PLANS	Assess the benefits of groups, classes, etc. consider other options	Communicate the potential benefits of attending groups, accessing resource
		Pass on examples of resource/groups/activities that have worked well or problems that have been overcome
		Reflect on how skills learnt in delivering PLANS have benefitted them (or not)

Overall, although the trial resulted in improved patient outcomes, PLANS received a mixed reception from participants. While some participants could see the personal and health benefits of engaging with PLANS and a recommended activity, others struggled to see the relevance of the intervention to them. Themes were identified which offer insight into the delivery and experience of PLANS from the perspective of telephone support workers and participants who received it and which offer understanding about the work that is entailed to engage with PLANS, to take action, and who is implicated in this process. These themes are: *formulation of ‘health’ in the context of everyday life; trajectories and tipping points: disrupting everyday routines; precarious trust in networks.*

### Formulation of ‘health’ in the context of everyday life

#### Problem? what problem?

A technology requires a problem to be defined that needs to be managed. This pre-condition was notable by its absence for some participants who were either unaware of having CKD or were unsure about the significance of the diagnosis which meant that PLANS (from the perspective of patients) often remained as a technology in search of a problem. Some participants recalled a very reassuring diagnosis of CKD in terms of it not being a problem and consequently felt unclear about whether they had a health problem and what, if anything, they should be doing about it. For example, one lady recalled a conversation with her nurse:

*‘she just said it’s just a slight bit of difference [i.e. in the results of a kidney function test], that’s all, I had to watch them….and it’s people with varying degrees of illness, she said, you haven’t actually got an illness, it’s just a change…which happens sometimes as you’re getting older’.* (White female 64 years)

This led to a perception that PLANS was irrelevant:

*‘Oh, it makes sense (PLANS), everything you’re doing makes sense......to me but I don’t actually need the actual what you’re offering, do you understand what I mean…I’m not trying to be funny….And as I say when (the baseline researcher) was here I just said to her I said you keep on about the condition and I said I’m not blagging but I haven’t got a condition at the minute’.* (White male 73 years)

There were even cases where participants reported that knowledge of CKD was as a result of participation in the trial and not through a discussion with a clinician. Thus engaging people with PLANS became a defacto means of disclosing a health diagnosis:

‘*Because the nurse, er, at the surgery suggested I do this (participate in the BRIGHT trial) …but I didn’t actually know there was anything wrong with me’.* (White female 69 years)

Rather than offering an additional form of support, finding out about a diagnosis of CKD through their participation in the study disrupted prior assumptions around their state of health. For example, one woman was concerned that problems with her kidneys which she became aware of via her participation in the trial might be related to the fact that her mother and two aunts died of stomach cancer:

*‘It’s something that’s always worried me, you know, erm, because you do……you know; my mother, and two of my aunties, died of the same thing, er, carcinoma of the stomach. And, you know, when you say the stomach you think kidneys, liver, you know, that sort of thing’.* (White female 64 years)

#### Shocked into action

However, health crises acted as a catalyst for a relatively young woman (48 years old), who whilst not typical, illustrates what was trying to be achieved with PLANS. This participant recalled a’ shock diagnosis’ of CKD after a health check at a local leisure centre and a subsequent appointment at a the renal unit. The apparent seriousness of her health led her to make connections with the death of her father who died young aged 56 and the shock of the diagnosis led to a re-evaluation of her life:

*‘Being ill and…or certainly having this condition just makes you readdress what you do and how you use your time’.* (Mixed-race female 48 years)

This participant became conscious of how work and family responsibilities had taken over her life and thus important for her to feel connected to her neighbours and community:

*‘It’s important to maintain those relationships and just maintain that connection with the community, with the people around you’.* (Mixed-race female 48 years)

The PLANS intervention had particular significance and it seemed that this shock diagnosis and associations with her deceased father led to an appraisal of what she was doing with her life and what was important to her:

*‘(The PLANS intervention) it’s awakened things in me that have always been there…I welcome the opportunity, because, like I say, you can live in a community and you don’t have a clue about what is around you…and some things can be so near and so accessible, but you just don’t know’.* (Mixed-race female 48 years)

#### Thinking beyond health: PLANS as a generic resource

Telephone support workers often encountered participants who were unclear about CKD and its health implications which contributed to confusion about the relevance of PLANS to participants. However, the telephone support workers displayed a good understanding of how PLANS was not specifically about supporting the management of CKD but was designed to open opportunities for discussion about areas of difficulty experienced by participants and confidently explored potential topics of more generic ‘real’ life importance to participants. The telephone support workers reported:

‘If there was no problem with the kidneys and they were very adamant there was no problem, therefore they needed no support, I’d think let’s try and get something practical out of this’. (TSW)

‘We’re finding that certain language, talking about health conditions…is difficult…and you just want to talk more generally about the problems that they’re facing that may be a result of their health, or may be the result of a whole bunch of other things’. (TSW)

The telephone support workers asked probing questions such as ‘what is most pressing for you at the moment?’ and ‘are you happy about that’ until they found some hints about what might be a helpful direction to steer the conversation.

Gathering background information about personal circumstances or significant health problems from participants helped to develop rapport and to encourage engagement with PLANS:

*‘I think it gave us an idea of what they might be interested in… and then that helped us with the questionnaire as well. Like if there was nothing that people were interested in, then we could obviously go back and use that information and say, well, you said this previously, um, is there anything else like that, that you might be interested in having a look at maybe’.* (TSW)

### Trajectories and tipping points: disrupting everyday routines

#### Lost confidence and acceptance of the status quo

It was evident that engagement with PLANS was not only influenced by understandings of health problems but also dependent on timing and the stage of life. Participants in the study trial were generally older and many reported losing touch with previously valued interests and pastimes. It was not uncommon for participants to report a gradual decline in their personal networks due to poor health, retirement or bereavement and this left many feeling emotionally and physically vulnerable and lacking in confidence about socialising or trying new things:

*‘Well, I used to have that, I used to have that, when I had the shop, I had the confidence, because people used to come in that weren’t very nice, you know, you had to be…stand up there and front them off, and things like that, I used to have the confidence, but, I think, as you stay at home, you lose your confidence, you lose your, erm, I can’t think of the right word…you become more fearful of people, or situations, in the sense of, well, what if I don’t fit in or, you know, I think, you have to just go, just not think about it, which is what I’m going to have to do, if I want to do something different’.* (White female 64 years)

This woman reported that life had become mundane and that her relationship with her husband was strained because of ‘being under each other’s feet all the time’. PLANS was relevant for her because it tapped into her feelings of needing to try new things and her concerns about being excluded from her bowling group. Regular childcare duties for her daughter and mobility problems limited her options but the conversation helped encourage her to see her husband as someone who could support her, particularly with transport, which encouraged a more positive perception of the relationship.

‘He (husband) takes me most of the places that I need to go… if it’s something that was, em, of interest then I think we’d probably both join, because my husband’s retired as well now’.

Similar feelings of withdrawal were reported by another participant whose role as a housewife and social anxiety problems had limited her social opportunities and had left her feeling lonely and depressed:

*‘I’ve become a recluse type person and this is not who I am, er, well, it’s not who we were. But he still goes out to work and I’ve got boreder and boreder and, um, more independent’.* (White female 61 years)

She told how she had developed low expectations of others regarding support:

TSW: ‘*Are you happy with that situation?’*

(White female 61 years) ‘*Sometimes I get very depressed about it. Sometimes I feel I could just… sit down and burst into tears’.*

The probing of the telephone support worker revealed interests in sewing, computer skills and participating in voluntary work:

*‘Yeah, that’s amazing that actually… I didn’t realise that I actually wanted to do the things that I actually mentioned to a [telephone support worker]……until she actually brought it up… You know, [the telephone support worker] actually reminded me of what we had before’.* (White female 61 years)

The telephone support workers told how it could be difficult to engage some of the older participants with PLANS because those who were well into their retirement generally reported being happy doing what they already did and were not interested in anything new. Alternatively participants who had more recently retired had a more engaged response to PLANS and they felt the intervention came at the ‘right’ time for them, because they were not yet set on any ideas about what they were going to do to fill their time. Therefore although PLANS was seen as relevant, engagement could be hindered by the long gap between retirement, things they used to enjoy, and the prospect of becoming involved in new social activities.

#### Appraisal of contexts and ‘where you are’

Health problems could be a major barrier to engagement, for example, some participants felt unable to take up any activities or services because of concerns about physical capability:

*‘There was other parts where actually like if they’ve got other health problems as well like, and they didn’t…they had less mobility and things like that as well, then you could obviously try and like bypass maybe exercise and physical activity, because they’d obviously said that they can’t like get out anywhere or move like properly’.* (TSW)

One participant with chronic fatigue syndrome described how she avoided participating in any regular social activities such as singing in the church choir or at social events (pastimes that she had previously enjoyed) because of feelings of vulnerability and concerns that she may ‘let others down’ if she was subsequently too unwell to attend. She told how occurrences of poor health had caused tensions in relationships with friends leaving her feeling isolation and depressed. However, talking to the telephone support worker and using PLANS appeared to have prompted a significant change in her outlook on life:

*‘Because I was getting where I felt I had no hope, truthfully and, I thought, this is my last…sum total of my life is sitting here now and just being ill and the days I can do a little, I, kind of, just fiddle around the house and I, kind of, was losing my identity, truthfully, and I just felt that she [i.e. the telephone support worker] was just giving me another network that’s out there, that I wasn’t aware of’.* (White female 57 years)

Since receiving the PLANS intervention she noted how she had driven the family car to pick up her grand-children from school, and how positive she felt about receiving a home visit from a hairdresser:

*‘…and I just feel, oh, somebody can actually come here who understands, who is sensitive to the condition, who I feel comfortable with and that will help me lift my spirits, because my world has just got out of control, because I can’t get out, simple thing like that to make me feel a bit better about myself’.* (White female 57 years)

PLANS gave an opportunity for participants to reflect on current circumstances and limitations and appraise the benefits of trying new things which required skills of the telephone support worker. The life trajectory of participants could in some cases act as a tipping point to action such as for retired participants who had begun to feel withdrawn and in some cases depressed. However, PLANS could also be seen as disrupting to everyday routines and challenging to normative assumptions about where participants were in life.

### Precarious trust in networks

#### The role of others in taking the first step

In some cases taking a ‘first step’ to making contact with groups or organisations was a difficult proposition which is perhaps why many just wanted to receive information. Even carers struggling to look after partners with poor health were reluctant to seek support because of a strong sense of personal responsibility, concerns that others ‘would not know what to do’, and because it may appear as though they were not coping leading to unwanted attention by external agencies.

One participant who had previously enjoyed strong community ties through involvement as a volunteer for her local hospital radio and organiser of a neighbourhood dance group revealed how difficult and isolating her life had become in recent years as her husband’s health had deteriorated. She was adamant she could not do anything that committed her to an appointment because of the unpredictable state of her husband’s health, and even going to see her GP regarding her own health was seen as problematic:

*‘I can book in and say I’m going, I could get up that morning, he’s not well enough to leave…So when you’re ringing up letting them down, and you’re thinking, some other person could have used that appointment….So you don’t book in again in case you have to do it again and…’*. (White female 60 years)

Unwillingness to seek support from family was not uncommon so as not to ‘burden’ them. Friends could play an important role in encouraging action. For example, one participant reported having been spurred on to try things by a friend of hers who said she would go with her:

*‘I found that the research that (the telephone support worker) did on that for me was, was quite informative because as soon as…it arrived, my friend arrived, and we were ticking off things that we were gonna do together…You know, (my friend) actually reminded me of what we had before’.* (White female 61 years)

Other participants who enjoyed a busy social life highlighted how friends or other relationships are important motivation for doing valued things, For example:

*‘I would be lost without friends in my life…and social things to do’.* (White female 71 years)

This was recognised by the support callers:

*‘For me, there were…cases that I found worked like that. Like they’d mentioned like only one specific friend or something like that, and then they’d…and the, um…the action planning section……they were saying, oh, well, I might take this friend with me……kind of thing, like when they came to that bit. So I think it does…it’s definitely a good question that one to ask people’.* (TSW)

Therefore sharing the experience with others was an important part of the process of engagement and action, allowed for an appraisal of the potential benefits, and lessened the anxiety of trying new things and meeting new people.

#### Fear of interference

However, the notion of receiving ‘outsider support’ seemed much less acceptable to some who felt that there were certain everyday tasks such as housework or odd jobs which were the province of family. Even if there was no family around, there was a strong sense of pride about not asking for help:

*‘It’s difficult. I think…I don’t know if it’s a generation thing or a cultural thing, but I think there’s still very much a family versus outsider support. And I think that was one of the difficulties was that we were recommending what people see as outsider support, so, you know, people come in and help you do the housework and, you know, patch up places in the house, that sort of thing. People feel like they should have family to do that for them, um, and actually they shouldn’t have to go outside for support like that. And I think the difficulty is when people lose that family support, because I think in some respects they were the most high risk group when family didn’t have time or when they didn’t have any family. Um, so it’s…it was a very sensitive one for…like so, for example, um, women that have just recently lost a husband, I think that was really difficult for them to ask for that outsider support to do a job that they feel has been done for them in the past, that sort of thing. So it was…I didn’t feel like you could really get to the bottom of it in an interview that was this, um, quick’.* (TSW)

One patient was horrified at the idea of being seen as a ‘scrounger’ which illustrates the social pressures of accepting help/interference from the state:

*‘There’s only my husband’s wages coming in. I don’t scrounge off the social or the dole, or anything like that; I never have done in all my married life. I don’t intend to start doing something I don’t believe in, you know. I know that issues with everybody are different. Mine just seem to be a bit OTT, my family, say, but there you go [laugh]. That’s the kind of person I am, I feel that, you know, you’ve got a purpose in life, go out and do it, don’t scrounge off anybody else in the doing because, you know, they have to scrounge off somebody else in the interim. It’s just…I don’t think people should be scrounging around, they should be helping themselves, um, which is exactly what I did. And if I can do it, I feel that there’s lots of other people out there that can do it too’.* (White female 61 years|)

There seemed to be embarrassment about being seen to need help with everyday things such as household chores which highlights the difficulties of offering support for people who don’t like the idea that they cannot cope. These are important reasons for not engaging with PLANS because some people don’t want outside interference or ‘meddling’ or they don’t want to be a burden on others. Self-reliant attitudes or the prioritisation of other things could also be challenging for the telephone support worker to engage participants with PLANS:

*‘And then, yes, it is something that I ought to be doing, and I know I ought to be doing but…I’ve got to…like last night, send an email to my brother…look at the finances, do this, do this…I have lists, endless lists of things to do……all the time. And it’s on the list, but I never seem to get down that far because there are more important, more immediate things…perhaps not more important, but more immediate things that have to be done’.* (White female 59 years)

#### Expectations of sociability

Some participants saw PLANS as only promoting social activity which could be an unwelcome intrusion. For example, one participant told how he was not a sociable person having preferred the solitary work of farming all his life. In response to information concerning local interest groups, he stated:

*‘Well, not really a great deal of interest in…I’m not really bothered. I’m not…I’m not a social bird really…(My wife’s) choir…they’re all very supportive, they all help one another. Whereas fellas will just walk away from that situation’.* (White male 81 years)

Another man felt that PLANS was suitable for people who have no friends or outside interests. His initial impression was that the suggestions made during the conversation with the telephone support worker regarding getting involved in social activities were unnecessary in his case:

*‘I felt it was a call more for people that lived on their own or didn’t have any friends or didn’t have any sort of connection with the outside world’.* (White male 73 years)

However resistant some participants were to the notion of PLANS, it was worth persevering in some cases where there may be a latent interest in doing something as happened with this participant who had taken up the suggestion of attending a computer course run at his local library. However, there remained staunch opposition from some participants to the idea of participating in any form of group activity, or the suggestion that improvement in wellbeing or health might be associated with increased social engagement. One participant said:

*‘I like being isolated, and I’d rather do my own thing… I don’t like depending on anybody….I never have… I’ve always done it myself and that’s it… I only ask somebody for help if I’m desperate’.* (White female 60 years)

Stoicism and resistance to the idea of anything ‘social’ or needing support meant support workers found they needed to be careful about how they presented themselves and the language they used in order to engage participants:

‘(the perception that) telling somebody they need to go to a group is almost like saying you don’t have a social life o…it’s almost like…judging them…making them a victim in a way’. (TSW)

The telephone support workers told how they found some men tricky to work with as they could be particularly resistant to the idea of anything social:

‘I think a lot of women had gone to groups in the past and they were alright with that, but I didn’t speak to a single man that would even contemplate the idea of going to any sort of what they perceived as a social group, they just were not having any of it’. (TSW)

## Discussion

PLANS has been developed as a self-management intervention which utilises local and community resources as a strategy to support people with vascular disease, its underlying ethos acknowledging the importance of the everyday contexts of living with a LTC and the range of problems experienced such as having access to everyday support, being active and involved in meaningful activities.

Resistance to the PLANS intervention ranged from an unclear health rationale for doing so, the double disruption of introducing a new intervention together with the unexpected knowledge that they were suffering from CKD, or the perception that their social life was under scrutiny. Men were particularly resistant to the PLANS intervention which is reflected in the difficulty recruiting them for this qualitative study (42% of the RCT participants were male). Men were more likely to see PLANS as an unwanted intrusion in their social life. It should also be noted that although the trial recruited from a wide geographical area of Greater Manchester including areas with high populations of ethnic minorities but very few participants in the trial were non-white (1.4%). It was therefore difficult to recruit non-white participants for this qualitative study with only one participant of mixed-race descent.

The invisibility or lack of awareness of CKD could be a hindrance to engagement with PLANS because some participants could not see the relevance of doing so and in some cases participants were upset because of the introduction of a new diagnosis. Therefore PLANS or any self-management resource is likely to struggle for relevance where there is an unclear health rationale for action. These findings suggest the need for greater consistency in the management of CKD within primary care for self-management support to be effective. It is reasonable to imagine that PLANS might have been more relevant or useful if delivered to a population with more ‘visible’ long-term health problems such as arthritis, heart disease or diabetes. However the ‘invisibility’ of CKD exposed some of the problems of delivering self-management support to people who do not prioritise their health in the context of other everyday life priorities and so demonstrates the value of an intervention like PLANS which operates at different levels, e.g. offering the opportunity to reflect on practical, personal or health-related problems which make life difficult but which have become normative.

It became clear that understanding the experiences of retirement was important for encouraging engagement with PLANS as was an appreciation of some of the hidden pressures and responsibilities older people encounter. Timing of PLANS is also important as growing older and retirement in circumstances where a LTC is involved appeared to risk a future where people lose touch with things they enjoy only to be faced with a further problem, that of finding it difficult to reconnect with others as a result of the passing of time and feelings of vulnerability.

The perception of ‘outsider support’ was another barrier to engagement with PLANS and demonstrates the difficulty of providing support for people who feel responsible for themselves and for those they look after. This illustrates the importance of understanding personal pride in relation to support, but also how feelings of vulnerability to unwanted attention by external agencies can influence engagement with PLANS. It seems that for some people who live day-to-day with overwhelming responsibilities, it is still regarded as better than available alternatives. As much of the sociological literature about chronic illness testifies, coming to terms with the onset of chronic health problems involves not just learning to manage clinical therapies, but also how this relates to valued aspects of life such as maintaining relationships and doing meaningful things.

For some participants, PLANS did not fit with the previous norms and sense of coherence of the work usually associated with a visit to primary care for a LTC. A focus on networks, socialising, participation, finding support and promoting activity as a means of alleviating the mundaneness of living life with a long-term condition sat uneasily with the normal expectations of healthcare support and advice on offer. For telephone workers, challenging everyday assumptions by offering alternatives to embedded routines required some skill, flexible thinking, and patience together with the ability to manage the unexpected realisation of the disclosure of a problem which many people were not aware they had.

Overall, PLANS involves work for patients. It requires engagement, rationalisation and understanding the need to do something else, and then taking the next step such as contacting groups or services. This process will likely involve negotiation with others and embedding into their lives. Hence there were occasions where participants only wanted information which was put ‘on the shelf’. Additionally the initial engagement with PLANS requires work from support workers who need the skills to encourage participation through finding trigger points for action and solutions to practical problems. This has implications for wider roll out as these are important factors for the effectiveness of a PLANS-style intervention.

One limitation of PLANS delivered in this way is that patients who are unable to use the phone adequately e.g. are hard of hearing, would not be able to receive the intervention in this format. This group are likely to be vulnerable and socially isolated who would benefit from an intervention to increase their social support and therefore it is important to consider how PLANS could be adapted as a face-to-face resource so that this group could access it.

NPT was an appropriate heuristic device with which to analyse this data as each of the four constructs relate well to the processes of engagement and the implementation of PLANS as experienced by participants in the study. Although we looked for data that fell outside of our coding framework, we were in fact able to code all relevant data with reference to one of the NPT constructs. However, whilst NPT is presented as a temporal process, this analysis showed that many participants experience the constructs of NPT simultaneously. For example, the work of sense-making necessarily involves an appraisal of the cost-benefits of PLANS for participants. Correspondingly, the work of engagement or ‘buy-in’ was influenced by these processes. This suggests that NPT is a useful way of understanding the experience of the PLANS intervention but best understood as a non-linear progression towards successful (or not) implementation.

## Conclusions

Improving awareness of and access to local resources offers a complementary approach to traditional individually-focused models of self-management which aim to increase the capacity and resources available to people with LTCs. Appreciation of the complex challenges faced by people in socially and economically deprived circumstances draws attention towards potentially valuable ways of supporting these groups. Whilst this study provides evidence for self-care strategies which consider the everyday life contexts in which health management takes place in order to tailor support, how it works in practice raises the relevance of how novel interventions based on this more social model lead to tensions both with the norms of practice operating in primary care (e.g. the non-disclosure of a diagnosis) and of people who have accommodated to a way of life in which isolation or the burden of competing activities has become normalised, as well as the dominance of a healthcare model where there is an underlying assumption (among both patients and professionals) that there is a sharp divide between illness management support and one’s broader well-being. Hence, the design and delivery of social interventions like PLANS need to take account of personal circumstances and commitments as a key priority, but it may need to also address issues of legitimacy, which may in turn require a closer and sustainable over time involvement by health professionals in the process of its delivery.

This study aimed to create understanding about pushing the boundaries of support that can be offered to people with LTCs to facilitate the adoption of this innovative and effective approach. Shifting the emphasis of self-management towards personal and community resources allows for building strategies which brings into the frame the utilisation of existing community, voluntary and third sector resources to support people with long-term health problems within socially disadvantaged communities. However, focussing on the everyday life contexts of health management raises debate about the need to address social and structural factors such as access to resources, available support, and home and work environment – but also suggests that the notion of engagement needs to be seen in the broader context of other agencies norms of practice and existing patient expectation.

## Abbreviations

PLANS: Patient-led assessment for network support; BRIGHT: BRinging information and guided help together; CKD: Chronic kidney disease; CLAHRC: Collaboration for leadership in applied health research and care; GPs: General practitioners; NPT: Normalization process theory; RCT: Randomized controlled trial.

## Competing interests

The authors declare that they have no competing interests.

## Authors’ contributions

CB with AK wrote the first draft of the paper. IV, CB, AK, AR, HB and PJ designed the original version of PLANS. CB, AK, AR, IV, HB, PJ, RM and TB conceived the study. CB, RB and HB collected the data. All authors contributed to the data analysis and writing the paper. All authors read and approved the final manuscript.

## Pre-publication history

The pre-publication history for this paper can be accessed here:

http://www.biomedcentral.com/1472-6963/14/262/prepub

## Supplementary Material

Additional file 1 Top 10 services sent to patients. Click here for file

Additional file 2 Interview questions for telephone support workers. Click here for file

Additional file 3 **NPT coding framework **[21]. Click here for file

Additional file 4 Participant demographics. Click here for file
