# Supplementary material for: Aligning everyday life priorities with people’s self-management support networks: an exploration of the work and implementation of a needs-led telephone support system
Source: BMC Health Serv Res. 2014 Jun 17;14:262. doi: 10.1186/1472-6963-14-262 (PMC4071856; doi:10.1186/1472-6963-14-262)
Supplement: Additional file 1 — Top 10 services sent to patients. [file 1472-6963-14-262-S1.docx]

**Additional file 1: Top 10 services sent to patients**

| Service/Group/Information | Total sent to patients |
| --- | --- |
| Patient Information Leaflet Dietetics and Nutrition Department (Healthy eating guide for people with kidney problems) | 42 |
| Salford U3A/ Salford Life Long Learning | 31 |
| Pensioners Link | 23 |
| The National Kidney Federation Helpline | 20 |
| NHS Choices website Kidney information (Not on PLANS) | 19 |
| Age UK Bolton | 13 |
| Age UK Learning and Resource Centre Farnworth | 9 |
| Guild Hall Community Centre | 9 |
| Sunshine House Community Centre | 9 |
| The British Kidney Patient Association (BKPA) | 9 |
