# Supplementary material for: Aligning everyday life priorities with people’s self-management support networks: an exploration of the work and implementation of a needs-led telephone support system
Source: BMC Health Serv Res. 2014 Jun 17;14:262. doi: 10.1186/1472-6963-14-262 (PMC4071856; doi:10.1186/1472-6963-14-262)
Supplement: Additional file 2 — Interview questions for telephone support workers. [file 1472-6963-14-262-S2.docx]

**Additional file 2:**

**Interview questions for telephone support workers**

| What are participants’ perceptions of the intervention?  Who is most likely to engage with the intervention?  Who is PLANS most suitable for?  What components of the intervention appear to promote positive engagement? (Discussion of biography, past activities, etc)  Clinical/Health: Do participants see the health benefits of PLANS or engage with the intervention for health reasons?  Social: Do participants see the benefits of social activity and recognise an opportunity to develop their personal networks?  What is the influence of current and previous engagement with activities on the uptake of PLANS recommendations?  What are inhibiting or supporting characteristics of participants? (e.g. isolated, family pressures or responsibilities)  How did participants engage with ideas for groups or activities?  How did the support worker effectively engage participants and arrive at a solution/group?  Do participants relate their health problems with the intervention or the groups/activities suggested?  What components of the package appear to resonate with participants in relation to managing their health?  What further support can be additional to this type of intervention?  Who is identified as potential support for engaging with new practices? |
| --- |

**Interview questions for intervention patients**

| What does the PLANS intervention mean to you?  Do you think the PLANS intervention is relevant to you?  What parts of the PLANS intervention do you remember most? (Discussion of biography, past activities, etc)  Clinical/Health: Do you see the health benefits of PLANS?  Social: Do you see the social benefits of PLANS?  Does the PLANS intervention remind you of things you have done in the past? Does it encourage you to try these things again or try something else?  What do you think helps or hinders you to try different things as a result of the PLANS intervention?  What do you think about your conversation with the telephone support worker? Did they/how did they help you find something you were interested in or found helpful?  Did you see the relevance of your PLANS results/recommendations in relation to your health?  What else do you think the PLANS intervention needs to be useful to you?  Who would help you do things for yourself? |
| --- |
