# Supplementary material for: Aligning everyday life priorities with people’s self-management support networks: an exploration of the work and implementation of a needs-led telephone support system
Source: BMC Health Serv Res. 2014 Jun 17;14:262. doi: 10.1186/1472-6963-14-262 (PMC4071856; doi:10.1186/1472-6963-14-262)
Supplement: Additional file 3 — NPT coding framework [21]. [file 1472-6963-14-262-S3.docx]

**Additional file 3: NPT coding framework** ([21](#_ENREF_21))

| **Normalisation Process Theory coding frame for evaluating PLANS** | | |
| --- | --- | --- |
| **NPT component** | **Questions to consider** | **Questions for PLANS analysis** |
| **Coherence**  **(i.e., meaning and sense making by participants)** | Is the intervention easy to understand and describe?  Is it clearly distinct from other interventions?  Does it have a clear purpose for all relevant participants?  Do participants have a shared sense of its purpose?  What benefits will the intervention bring and to whom?  Are these benefits likely to be valued by potential participants? | How do the telephone support workers describe the intervention?  Is there a ‘lightbulb’ moment when participants understand the personal relevance of the intervention?  In what way is the intervention relevant to them? |
| **Cognitive participation**  **(i.e., commitment and engagement by participants)** | Are participants likely to think it is a good idea?  Will they see the point of the intervention easily?  Will they be prepared to invest time, energy and work in it? | Describe the processes of engagement.  Beyond ‘rapport’, how do the support workers engage participants? |
| **Collective action**  **(i.e., the work participants do to make the intervention function)** | How will the intervention affect the work of user groups?  How will doing PLANS activities impact on or enhance relationships with others in their social network? | Who is identified as important to realise the individual goals of participants?  Does the PLANS intervention implicate ‘work’ for others (friends, self, family)?  What is the work done by each aspect of the intervention? E.g. what does the telephone support do, what does the PLANS questionnaire do?  How much work does the support worker have to do to make the intervention work? E.g. finding acceptable and relevant resources and encouraging uptake. |
| **Reflexive Monitoring**  **(i.e., participants reflect on or appraise the intervention** | How are users likely to perceive the intervention once it has been in use for a while?  Can the intervention be adapted or improved on the basis of experience? | At follow up and interview, how have participants understood the intervention and how do they plan to integrate new activities into their lives?  How could the intervention be delivered more effectively or tailored for specific groups? |
