# Supplementary material for: Aligning everyday life priorities with people’s self-management support networks: an exploration of the work and implementation of a needs-led telephone support system
Source: BMC Health Serv Res. 2014 Jun 17;14:262. doi: 10.1186/1472-6963-14-262 (PMC4071856; doi:10.1186/1472-6963-14-262)
Supplement: Additional file 4 — Participant demographics. [file 1472-6963-14-262-S4.docx]

**Additional file 4: Participant demographics**

| **Patient number** | **Gender** | **Ethnicity** | **Age** | **Total number of conditions** |
| --- | --- | --- | --- | --- |
| 421 | Female | White British | 64 | 5 |
| 464 | Female | White British | 69 | 4 |
| 473 | Female | White British | 61 | 6 |
| 477 | Female | White British | 60 | 2 |
| 482 | Male | White British | 73 | 2 |
| 506 | Male | White British | 71 | 4 |
| 511 | Female | White British | 78 | 4 |
| 515 | Female | White British | 71 | 2 |
| 521 | Female | White British | 59 | 5 |
| 524 | Female | White British | 61 | 2 |
| 532 | Female | White British | 72 | 1 |
| 547 | Female | White British | 80 | 3 |
| 558 | Male | White British | 81 | 5 |
| 560 | Female | Mixed-race | 48 | 2 |
| 566 | Female | White British | 57 | 5 |
| 602 | Female | White British | 69 | 3 |
| 618 | Female | White British | 60 | 5 |
| 628 | Female | White British | 71 | 5 |
| 629 | Male | White British | 84 | 1 |
| 634 | Male | White British | 90 | 4 |
